# Rejuvenated iPSC-derived GD2-directed CART Cells Harbor Robust Cytotoxicity Against Small Cell Lung Cancer

**DOI:** 10.1158/2767-9764.CRC-23-0259

**Published:** 2024-03-11

**Authors:** Shintaro Kinoshita, Midori Ishii, Jun Ando, Takaharu Kimura, Tomoyuki Yamaguchi, Sakiko Harada, Fumiyuki Takahashi, Kazutaka Nakashima, Yozo Nakazawa, Satoshi Yamazaki, Koichi Ohshima, Kazuhisa Takahashi, Hiromitsu Nakauchi, Miki Ando

**Affiliations:** 1 Department of Hematology, Juntendo University School of Medicine, Tokyo, Japan.; 2 Division of Cell Therapy and Blood Transfusion Medicine, Juntendo University School of Medicine, Tokyo, Japan.; 3 Laboratory of Stem Cell Therapy, Faculty of Medicine, University of Tsukuba, Ibaraki, Japan.; 4 Laboratory of Regenerative Medicine, Tokyo University of Pharmacy and Life Science, Tokyo, Japan.; 5 Department of Respiratory Medicine, Juntendo University School of Medicine, Tokyo, Japan.; 6 Department of Pathology, School of Medicine, Kurume University, Fukuoka, Japan.; 7 Department of Pediatrics, Shinsyu University School of Medicine, Nagano, Japan.; 8 Institute for Stem Cell Biology and Regenerative Medicine, Stanford University School of Medicine, Stanford, California.; 9 Stem Cell Therapy Laboratory, Advanced Research Institute, Tokyo Medical and Dental University, Tokyo, Japan.

## Abstract

Small cell lung cancer (SCLC) is exceptionally aggressive, with limited treatment options. Disialoganglioside (GD2) is highly expressed on SCLC and is considered a good target for chimeric antigen receptor (CAR) T cells (CART). Although GD2-directed CARTs (GD2-CART) exhibit cytotoxicity against various GD2-expressing tumors, they lack significant cytotoxicity against SCLC. To enhance cytotoxicity of GD2-CARTs against SCLC, we introduced GD2-CAR into induced pluripotent stem cells (iPSC)-derived rejuvenated cytotoxic T lymphocytes (GD2-CARrejT). GD2-CARrejTs acted much more strongly against SCLC cells than did GD2-CARTs both *in vitro* and *in vivo*. Single-cell RNA sequencing elucidated that levels of expression of *TIGIT* were significantly lower and levels of expression of genes associated with cytotoxicity were significantly higher in GD2-CARrejTs than those in GD2-CARTs. Dual blockade of TIGIT and programmed death-1 (PD-1) increased the cytotoxicity of GD2-CARTs to some extent, suggesting that low TIGIT and PD-1 expression by GD2-CARrejTs is a major factor required for robust cytotoxicity against SCLC. Not only for robust cytotoxicity but also for availability as “off-the-shelf” T-cell therapy, iPSC-derived GD2-CARrejTs are a promising novel treatment for SCLC.

**Significance:**

This research introduces iPSC-derived rejuvenated GD2-CARTs (GD2-CARrejT) as a novel approach to combat SCLC. Compared with conventional GD2-CARTs, GD2-CARrejTs with reduced TIGIT and PD-1 expression demonstrate robust cytotoxicity against SCLC and would be a promising therapy for SCLC.

## Introduction

Small cell lung cancer (SCLC) represents approximately 15% of all lung cancers (1). Its high proliferative rate and early metastasis (2) mean that 80%–85% of patients with SCLC at diagnosis have extensive-stage disease (3). Although by far most patients respond to initial chemotherapy, relapse within 6 months is usual, with 10–13 months median overall survival (4). Because the prognosis is devastating, the U.S. NCI designates SCLC as a recalcitrant cancer (5). Before FDA approval of anti-programmed death ligand 1 (PD-L1) antibody, no new treatment for SCLC had appeared in over three decades. However, anti-PD-L1 antibody achieves response in only a small subset of patients (6, 7). New treatment strategies for SCLC thus are urgently needed.

Disialoganglioside (GD2) is extracellularly expressed on tumors of neuroectodermal origin such as neuroblastoma (85%–100%; refs. 8–11), retinoblastoma (100%; ref. 12), osteosarcoma (88%–100%; ref. 13), melanoma (50%; refs. 14, 15), Ewing sarcoma (40%–90%; ref. 16), and SCLC (80%; ref. 17). Although anti-GD2 mAbs improve outcome over that with conventional chemotherapy in neuroblastoma, at least 40% of patients with neuroblastoma given anti-GD2 mAbs relapsed (18, 19). Moreover, despite broad expression of GD2 on osteosarcoma, anti-GD2 antibodies did not show significant antitumor activity in osteosarcoma or any other GD2-expressing cancers, including SCLC (18, 19). Chimeric antigen receptor (CAR) T cells (CART) can induce durable remission in relapsed and refractory hematologic malignancies (20, 21). GD2 is an attractive target for CART therapy targeting GD2-expressing tumors; several phase I clinical trials of GD2-directed CART therapy in neuroblastoma demonstrated safety and efficient *in vivo* expansion without toxicities of consequence (22–24). However, reports of clinical GD2-CART therapy in SCLC are lacking.

We thus have developed GD2-CART therapy targeting SCLC. We first generated lentiviral GD2-CARTs from peripheral blood. Although these killed GD2-expressing tumors including neuroblastoma, glioblastoma, Ewing sarcoma, and lymphoma, GD2-CARTs exhibited only very slight cytotoxicity against SCLC lines.

To reprogram exhausted antigen-specific CTLs into iPSCs and to differentiate the iPSCs into functionally rejuvenated CTLs (rejuvenated CTLs; rejT) yields rejTs with cytotoxicity against several refractory tumors greater than that of original CTLs (25–28). Cytotoxicity of Epstein-Barr virus (EBV) latent membrane protein 2 (LMP2)-specific rejTs (LMP2-rejT) is more robust than that of original CTLs, with remarkable persistence for > 7 months *in vivo* (29). This approach successfully generates dual antigen receptor rejTs that can recognize two antigens, one via CAR and the other via native T-cell receptor. These dual antigen receptor rejTs potently suppress EBV-associated lymphomas more efficiently than do single antigen receptor CTLs (30). To enhance the cytotoxicity of GD2-CARTs against SCLC, we introduced GD2-CAR into LMP2-rejTs, which persist robustly *in vivo* (29). We then examined the antitumor effect of GD2-CARrejTs against SCLC.

## Materials and Methods

### Cell Lines

Among SCLC cell lines, SCLC-J1 was established in our laboratory from pleural-effusion fluid of a patient with SCLC (31). Lines NCI-H82 (RRID: CVCL_1591), NCI-H69 (RRID: CVCL_1579), and NCI-H446 (RRID: CVCL_1562) were purchased from ATCC. All four SCLC cell lines were cultured in RPMI1640 medium (RPMI; catalog no. R8758; Thermo Fisher Scientific) supplemented with 10% FBS (catalog no. 10270106; Gibco Life Technology) and 1% penicillin-streptomycin-glutamine (PSG; catalog no. 10378-016, Thermo Fisher Scientific). To evaluate antitumor effect of GD2-2840z-CARTs and GD2-CARrejTs on SCLC, NCI-H446 cells were transduced with a γ-retroviral vector encoding both firefly luciferase (FFluc) and GFP to monitor tumor volume. GFP-expressing cells were sorted by flow cytometry and maintained in RPMI supplemented with 10% FBS and 1% PSG. The non–small cell lung cancer cell line A549 (RRID:CVCL_0023) was purchased from ATCC and cultured in DMEM (catalog no. D5796, Thermo Fisher Scientific) supplemented with 10% FBS and 1% PSG. The GD2-expressing extranodal natural killer/T-cell lymphoma, nasal type, cell line NK-YS was a gift from Dr. J. Tsuchiyama (Okayama University Medical School, Okayama, Japan). NK-YS cells were grown in Iscove's modified Dulbecco's medium (IMDM; catalog no. 13390, Thermo Fisher Scientific) supplemented with 10% FBS, 1% PSG, and 100 IU/ mL IL2 (catalog no. 130-097-743, Miltenyi Biotec). Neuroblastoma cell line LA-N-5 (RRID: CVCL_0389) and glioblastoma cell line T98G (RRID: CVCL_0556) were purchased from RIKEN BRC and were grown in RPMI supplemented with 10% FBS and 1% PSG. We did not perform *Mycoplasma* testing and authentication for SCLC-J1 and NK-YS cell lines. The LA-N-5 and T98G cell lines, being recently acquired, were not subjected to these checks either. However, for other cell lines purchased directly from ATCC, *Mycoplasma* testing was conducted immediately after purchase, and all tested negative. To differentiate GD2-CARrejTs from iPSCs, C3H10T1/2 feeder cells (RCB0247, RIKEN BRC) and C3H10T1/2 feeder cells expressing the human homologs of two Notch Delta ligands, delta-like1 (DL1) and delta-like4 (DL4), were used. C3H10T1/2 cells were transduced with a retrovirus encoding both DL1 and DL4. These feeder cells were cultured in minimum essential medium α (MEMα; catalog no. 12571063, Thermo Fisher Scientific) supplemented with 10% FBS and 1% PSG. The HEK 293T cell line (RCB2202, RIKEN BRC) for lentivirus or retrovirus production was maintained in DMEM supplemented with 10% FBS and 1% PSG.

The experimental protocols were approved by the Research Ethics Committee for the Faculty of Medicine, Juntendo University (Bunkyo-ku, Tokyo, Japan), and were in accordance with the Declaration of Helsinki. Written informed consent was obtained from healthy donors and patients.

### Vector Constructs

Retroviral SFG-iCaspase9 (iC9)-14G2a (GD2)-CD28-OX40z plasmid was a gift from Dr. M.K. Brenner (Baylor College of Medicine, Houston, TX).

To generate a lentiviral Cs-Ubc-GD2-CD28-OX40-CD3z-F2A-mCherry construct, GD2-CD28-OX40-CD3z was amplified by PCR from the retroviral SFG-iC9-GD2-CD28-OX40-CD3z plasmid and fused with a lentiviral Cs-Ubc-iC9-F2A-mCherry construct (25) by replacing iC9 with GD2-CD28-OX40-CD3z using InFusion cloning (Takara Bio).

To compare the effectiveness of 4-1BB, CD28, and CD28-OX40 costimulatory domains, we cloned a Cs-Ubc-GD2-CD28-F2A-mCherry construct and a Cs-Ubc-GD2-4-1BB-F2A-mCherry construct. The 4-1BB intracellular costimulatory domain was amplified by PCR from a retroviral CD19-CAR plasmid given by Dr. C Imai (Niigata University, Niigata, Japan; ref. 30) and fused with the lentiviral Cs-Ubc-GD2-CD28-OX40-CD3z-F2A-mCherry vector construct by replacing CD28-OX40 with 4-1BB using InFusion cloning. GD2-CD28 was amplified by PCR from the Cs-Ubc-GD2-CD28-OX40-CD3z-F2A-mCherry vector construct and fused with the lentiviral Cs-Ubc-GD2-CD28-OX40-CD3z-F2A-mCherry vector construct by replacing CD28-OX40 with CD28 using InFusion cloning.

### Virus Production

To produce retroviral supernatant, 293T cells were cotransfected with three plasmids [the retroviral construct; Peg-Pam (encoding *gag-pol*); RDF (encoding RD114 envelope)] using Gene Juice transfection reagent (Sigma-Aldrich). Supernatants were collected 48 hours after transfection (32). To produce a lentiviral supernatant, 293T cells were cotransfected using Ca_2_PO_4_ with three plasmids (the lentiviral construct; pMDLg/pRRE plasmid; pCMV-VSV-G-RSV-Rev plasmid) as described previosuly (25). Virus supernatants were collected 48–72 hours later.

### Peripheral Blood–derived CART Generation

Peripheral blood mononuclear cells (PBMC) of healthy donors were activated by T Cell TransAct (Miltenyi Biotec) in the presence of IL2 (100 U/mL). Three days later, activated T cells were plated onto 24-well plates coated with RetroNectin (Takara Bio) and lentiviral GD2-CAR supernatant was added to each well. GD2-CARTs were cultured in NS-A2 (catalog no. IW-66001, Nissui) with 10 ng/mL each of IL7 (catalog no. 130-095-367) and IL15 (catalog no. 130-095-760; both Miltenyi Biotec). We named generated GD2-CARTs after their costimulatory domains: GD2-2840z-CARTs, GD2-28z-CARTs, and GD2-BBz-CARTs. CAR-positive T cells were sorted so that CAR transgene expression in GD2-CARTs was approximately 80% to minimize independent variance.

### CAR Transduction into iPSCs

iPSCs derived from an LMP2-CTL clone (TYGPVFMSL; ref. 30) were transduced with CAR lentiviral vector (multiplicity of infection, 20) in wells coated with vitronectin (catalog no. A14700; Thermo Fisher Scientific) and were cultured in Essential 8 medium (catalog no. A2656101, Thermo Fisher Scientific). On day 7, cells were sorted (RRID: SCR_018893, MoFlo Astrios EQs; Beckman Coulter) for transduced iPSCs expressing mCherry.

### Differentiation of GD2-CAR-iPSCs into GD2-CARrejTs

GD2-2840z-CAR-iPSCs were cultivated on C3H10T1/2 feeder cells in IMDM supplemented with 15% FBS (catalog no. SH30070, HyClone, GE Healthcare UK) and a cocktail of 10 mg/mL insulin, 5.5 mg/mL transferrin, 5 ng/mL sodium selenite, 2 mmol/L l-glutamine (catalog no. 41400045; all Thermo Fisher Scientific), 0.45 mmol/L a-monothioglycerol (catalog no. 155723, Sigma-Aldrich), and 50 mg/mL ascorbic acid (catalog no. 87314, Takeda Pharmaceuticals) in the presence of 20 ng/mL VEGF (catalog no. 130-109-386, Miltenyi Biotec). On day 14 of coculture, sac-like structures containing hematopoietic progenitor cells were extracted and transferred onto C3H10T1/2 feeder cells expressing Delta-like ligands 1 and 4 in MEMα supplemented with FBS (HyClone) in the presence of 20 ng/mL human stem cell factor, 10 ng/mL human Fms-related tyrosine kinase 3 ligand (catalog no. 130-096-474), and 10 ng/mL IL7 (all Miltenyi Biotec). On day 28 of coculture, T-lineage cells were harvested and stimulated with irradiated PBMCs in NS-A2 in the presence of 5 mg/mL phytohemagglutinin (catalog no. 11249738001, Sigma-Aldrich) and 10 ng/mL IL7 and IL15 (both Miltenyi Biotec).

### Antibodies

To confirm GD2 expression on SCLC cells, we used allophycocyanin-conjugated (APC) anti-human ganglioside GD2 clone 14G2a (RRID: AB_2563083; BioLegend) and APC mouse IgG2a, κ isotype, control antibody clone MOPC-173 (RRID: AB_326468; BioLegend). To examine GD2-CAR transgene expression, we used biotin-SP–conjugated AffiniPure goat anti-mouse IgG, F(ab')2 fragment specific (RRID: AB_2338565; Jackson ImmunoResearch) followed by APC-streptavidin (RRID: AB_10050396; BD Biosciences). To examine T-cell subsets and antigen specificity via flow cytometry (as described below), we used PE-conjugated HLA-A*2402/TYGPVFMSL MHC tetramer (catalog no. TS-M035-1, MBL), APC/cyanin-7–conjugated (C7) mouse anti-human CD3 clone HIT3a (RRID: AB_314054), APC mouse anti-human CD4 clone OKT4 (RRID: AB_571945), and Pacific-Blue–conjugated (PB) mouse anti-human CD8a clone RPA-T8 (RRID: AB_2174122; all BioLegend). To characterize GD2-expressing tumors, we used Brilliant-Violet–conjugated (BV) 421 anti-human CD155 clone SKII.4 (RRID: AB_2810525) and fluoresceinisothiocyanate isomer-I anti-human CD274 (B7-H1, PD-L1) antibody clone MIH2 (RRID: AB_2734471; both BioLegend). To examine expression of exhaustion markers via flow cytometry, we used BV 605 anti-human CD223 (LAG-3) antibody clone 11C3C65 (RRID: AB_2721540), APC mouse anti-human programmed death-1 (PD-1) clone EH12.2H7 (RRID: AB_940473), and APC/Cy7 anti-human CD366 (Tim-3) antibody clone F38-2E (RRID: AB_2565716; all BioLegend). To confirm cytotoxic potentials of GD2-CARTs and GD2-CARrejTs, both underwent intracellular immunostaining for granzyme B and perforin. The cells were cultured *in vitro* for 14 days after stimulation with 5 µg/mL PHA-L (Sigma-Aldrich) mixed with irradiated PBMCs in NS-A2 CTL medium (Nissui) supplemented with 1% PSG in the presence of 10 ng/mL IL7 and 10 ng/mL IL15 (both Miltenyi Biotec). We used PE/Cy7 anti-human perforin clone B-D48 and PB anti-human/mouse granzyme B antibody. To examine expression of TIGIT and CD226 on T cells, we used PE anti-human TIGIT, clone A15153G (RRID: AB_2632729), and APC anti-human CD226 antibody, 11A8 (RRID: AB_2561951; both BioLegend).

### Flow Cytometry

Flow cytometry was performed on BD FACSAria II (RRID:SCR_018934) or BD LSRFortessa equipment (RRID: SCR_023638, BD Biosciences). The acquired data were analyzed using FlowJo software 10.5.3 (RRID:SCR_008520; Tree Star). Propidium iodide or 7-aminoactinomycin D (catalog no. P1304MP) was used to gate in live cells in all analyses. A fluorescence-minus-one technique was used to interpret flow cytometry data in all antibody combinations.

### Chromium-51 Release Assay

Chromium-51 (^51^Cr) release assays were carried out to evaluate cytolysis by GD2-2840z-CARTs and GD2-CARrejTs. Target cells were preincubated with ^51^Cr for 1 hour and then cocultured for 6 hours with effector cells at effector: target ratios (E:T ratios) from 40:1 to 5:1 (33). Target cells incubated in complete medium without adding effector T cells or 0.2% TritonX-100 (catalog no. A16046-AE, Thermo Fisher Scientific) were used to determine spontaneous and maximum ^51^Cr releases, respectively. Percentages of cytolysis were calculated as [(experimental release − spontaneous release)/(maximum release − spontaneous release)] × 100 (%).

### Impedance-based Tumor Killing Assay

Cytotoxicity assays using xCELLigence real-time cell analyzers [RRID:SCR_014821; real-time cell analysis (RTCA), ACEA Biosciences] were performed to verify tumor-suppressive effect for both GD2-2840z-CARTs and GD2-CARrejTs. We cocultured target cells (H446 or H69) with control T cells, GD2-2840z-CARTs, and GD2-CARrejTs. Target cells (H446: 4 × 10^4^ cells per well, H69: 2 × 10^4^ cells per well) were seeded and cultured for 35 hours (H446) and 42 hours (H69) in microelectrode-coated 96-well plates to permit target cell adherence. Effector T cells were then added (E:T 1:1). Electrical impedance changes were recorded automatically and continuously as cell index (CI) by RTCA every 15 minutes for up to 150 hours.

We also evaluated the antitumor efficacy of GD2-2840z-CARTs with anti-PD-1 antibody (catalog no. BE0146, InVivoMAb anti-human PD-1, BioXCell) and anti-TIGIT antibody (Tiragolumab, Seleck Biotech) against SCLC using RTCA. The samples were divided into four groups: a target only group, single blockade by anti-PD-1 antibody group, single blockade by anti-TIGIT antibody group, and dual blockade by anti-PD-1 and TIGIT antibodies group. H446 cells (3 × 10^4^ cells per well) were seeded and cultured for 18.5 hours in microelectrode-coated 96-well plates to permit target cell adherence. Anti-PD-1 antibody was added at a final concentration of 20 µg/mL on coculture days −4, −1, and 0, while anti-TIGIT antibody was added at a final concentration of 20 µg/mL on coculture days −1 and 0. Effector T cells were then added (E:T ratio, 1:2). Electrical impedance changes were recorded automatically and continuously as CI by RTCA every 15 minutes for up to 100 hours.

Data were analyzed and graphed using the xCELLigence RTCA software package (version 2.0). 0.2% TritonX-100 (Thermo Fisher Scientific) was added to some wells to determine a complete cell lysis index (CI_max_). Percentages of killing (% killing) were calculated as ([CI_no effector_ − CI_effector_]/[CI_no effector_ − CI_max_]) x 100 (%).

### Analysis of Cytokine Release Capacity Through Cytometric Bead Array

To determine the concentrations of the cytokines released by GD2-CARTs or GD2-CARrejTs during coculturing, cytokines IL2, IL4, IL6, IL10, TNF, IFNγ, and IL17A in culture medium were measured using the BD Cytometric Bead Array (CBA) Human Th1/Th2/Th17 Cytokine Kit (RRID:AB_2869353, BD Biosciences) according to the instructions provided by the manufacturer. GD2-CARTs (1 × 10^5^ cells) or GD2-CARrejTs (1 × 10^5^ cells) were cocultured with SCLC-J1 (1 × 10^5^ cells) in 96-well plates for 16 hours, with collection of supernatant samples. Levels of multiple cytokines were simultaneously determined by flow cytometry (LSRFortessa, BD Biosciences). The obtained data were analyzed using CBA software (catalog no. 550065, BD Biosciences).

### Antitumor Activity, *In Vivo* Model

All *in vivo* studies were approved by the Animal Research Committees of Juntendo University School of Medicine (Tokyo, Japan). To verify the tumor-suppressive effect of GD2-CARrejTs against SCLC, the H446 SCLC cell line was transduced with a γ-retroviral vector encoding a fusion protein composed of GFP and FFluc. GFP/FFluc H446 cells (1 × 10^6^ cells/mouse) were injected intravenously into 6-week-old female nonobese diabetic/Shi-severe combined immunodeficiency, IL2Rγ knockout Jic (NOG) mice (In-Vivo Science) and tumor growth was monitored using the Caliper *in vivo* imaging system (Caliper Life Sciences). Firefly d-luciferin substrate (OZ Biosciences) was intraperitoneally injected into mice 15 minutes before imaging. Mice were divided into three groups: no-treatment group, GD2-2840z-CART therapy treated group, and GD2-CARrejT treated group. Four days after tumor inoculation, mice were intravenously treated with GD2-2840z-CARTs or GD2-CARrejTs (1.0 × 10^7^ cells). Living Image software version 4.7.2 (RRID:SCR_014247; PerkinElmer) was used for luminescence analyses. The intensity of signal was measured as total photon/s/cm^2^/steradian (p/s/cm^2^/sr).

### qRT-PCR

Genomic DNA was isolated from samples of mouse whole blood and from cultured GD2-CARTs using a QIAamp DNA blood mini kit (catalog no. 51183, QIAGEN). GD2-CAR vector copies were quantified using the StepOnePlus real-time PCR system (RRID:SCR_015805; Applied Biosystems). Primers and probes for the GD2-CAR transgene and *GAPDH* were custom-ordered (Applied Biosystems) as described previously (34). Individual PCR reaction results were normalized against GAPDH levels. Copies of transgene/µg of DNA were quantified as copies calculated from GD2 standard curve/input DNA (ng) correction factor (ng detected/ng input) 1,000 ng, as described previously (35). Table 1 provides primer and probe sequences.

**TABLE 1:** Primer and probe information

Primer and probe	Sequence
Forward	5′-GCTGCACCAACTGTATCCATCTT-3′
Reverse	5′-GGTCCAGACTGCTGAAGCT-3′
Probe	5′-CACCCGACCCACCACC-3′

### IHC Staining and Image Capture

Mouse tissue samples were fixed in phosphate-buffered aqueous 4% paraformaldehyde solution (catalog no. 161-20141, Wako Pure Chemicals). Sections of paraffin-embedded tissues, picked up on glass slides, were stained with hematoxylin and eosin for histopathologic examination. Anti-human CD3 rabbit mAb (SP7; 1:50 dilution; ab16669, Abcam, RRID:AB_443425) was used for immunostaining (29, 36–38). Tissue sections were deparaffinized with xylene and ethanol and then rehydrated with H_2_O. To recover antigen, slides were heated in a microwave oven at 95°C for 20 minutes in ethylenediaminetetraacetic acid aqueous buffer solution (pH 8.0). After cooling and washing with buffer solution, the slides were treated using a Dako autostainer (Dakocytomation). Endogenous peroxidase activity was blocked by 5 minutes’ incubation in 3% H_2_O_2_. The slides were incubated with antibody at room temperature in a humidified chamber for 30 minutes and then incubated with the Dako REAL EnVision Detection System horseradish peroxidase–conjugated anti-rabbit secondary antibody (K4003; Dakocytomation) at room temperature for 30 minutes. Reaction products were visualized by treatment with diaminobenzidine chromogen (Dakocytomation) for 5 minutes. Immunohistopathologic images were obtained using a BX53 microscope (Olympus).

### Library Preparation for Single-cell RNA Sequencing and Cellular Indexing of Transcriptomes and Epitopes by Sequencing

Approximately 1 × 10^6^ sample cells (*in vitro* cultured GD2-2840z-CARTs and GD2-CARrejTs, obtained 14 days after stimulation with 5 µg/mL PHA-L (Sigma-Aldrich) mixed with irradiated PBMCs in NS-A2 CTL medium (Nissui) supplemented with 1% PSG in the presence of 10 ng/mL IL7 and 10 ng/mL IL15 (both Miltenyi Biotec), were resuspended with 50 µL of chilled PBS + 0.04% BSA (Thermo Fisher Scientific). CAR transgene expression in GD2-CARTs uniformly was approximately 80%, as CAR-positive T cells were sorted before using the assay. Samples were labeled with TotalSeq-B Human Universal Cocktail, V1.0 (RRID: AB_2892472, BioLegend), an antibody cocktail conjugated with a unique identifier in the form of a barcode and used for tracking expression profiles.

After staining, cells were washed four times to remove residual antibodies. The labeled cells were then loaded onto the 10x Chromium system with the Chromium Next Gem Single Cell 3′ Reagent Kits v 3.1 (Dual Index; catalog no. PN-1000128, 10x Genomics). Libraries generated from cell surface protein labeling with TotalSeq-B antibodies were sequenced (Macrogen). The Shirokane supercomputer system and Cell Ranger (RRID:SCR_017344, version 7.0.0, 10x Genomics) were used for demultiplexing and alignment (human reference genome GRCh38). Downstream analyses used Seurat (v4.0; ref. 39). Doublet Finder was used to remove doublets (40) and samples were filtered for cells with < 15% mitochondrial gene expression in which >200 genes were detected per cell. Samples were normalized using NormalizeData and ScaleData followed by integration of samples with clustering and Uniform Manifold Approximation and Projection (UMAP) analysis using FindMultiModalNeighbors and RunUMAP. Control T cells were obtained from 10k PBMCs from a Healthy Donor-Gene Expression and Cell Surface Protein [PBMC, 10k; Cellular Indexing of Transcriptomes and Epitopes by Sequencing (CITE-seq); https://support.10xgenomics.com/single-cell-gene-expression/datasets/3.0.0/pbmc_10k_protein_v3 (2018)].

### Statistical Analysis

All data are presented as mean ± SD or SEM. Results were analyzed by unpaired Student *t* test (two-tailed) or ANOVA as stated, with a *P* value <0.05 indicating statistical significance. Survival curves were compared using Kaplan–Meier analysis with log-rank testing. Software for all statistical analyses was Excel (Microsoft) and Prism 9.0 (RRID:SCR_002798; GraphPad Software).

### Data Availability

The single-cell RNA sequencing (scRNA-seq) data of the GD2-2840z-CARTs and GD2-CARrejTs are deposited in the Gene Expression Omnibus database with accession number GSE245955.

## Results

### GD2-2840z-CARTs Expressed Exhaustion Markers at Levels Lower than those in GD2-28z-CARTs and GD2-BBz-CARTs

**FIGURE 1:**
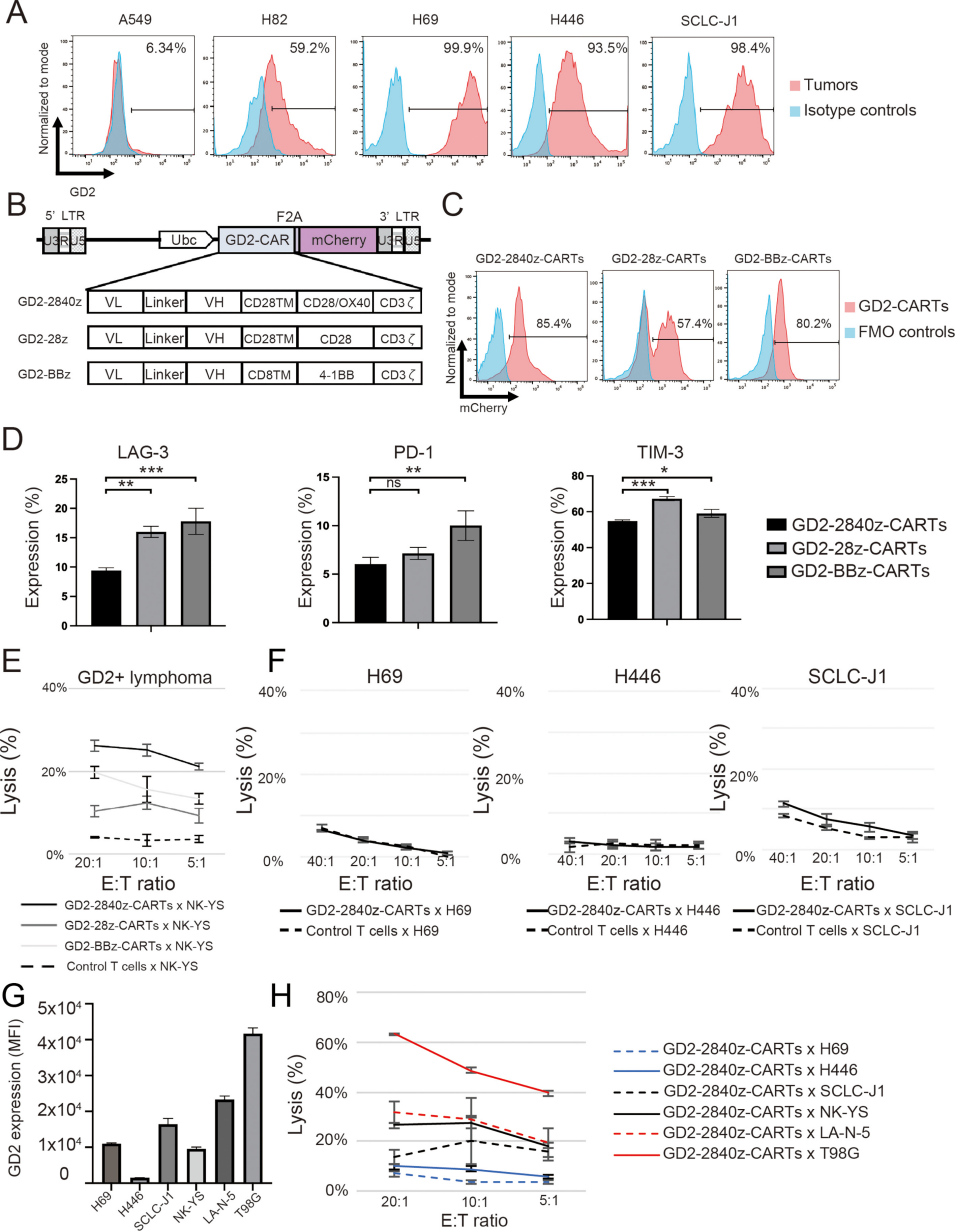
Generation of GD2-CARTs. **A,** GD2 expression by NSCLC cell line (negative control) and by four SCLC cell lines (red), with isotype control (blue). The plots represent three independent experiments. **B,** Schema, lentiviral GD2-CAR-vectors containing Ubc promoter. CAR contains an scFv derived from heavy (VH) and light chain (VL) variable regions of 14G2a mAb. TM, transmembrane. Each CAR has a costimulatory domain (highlighted in red): GD2-2840z, CD28 and OX40; GD2-28z, CD28; and GD2-BBz, 4-1BB. The constructs contain the cleavable 2A-like sequence and mCherry. **C,** Flow cytometric analysis, GD2-CAR transgene expression in GD2-2840z-CARTs, GD2-28z-CARTs, and GD2-BBz-CARTs (red). Fluorescence minus one (FMO) control (blue). The histogram represents three independent experiments. **D,** Flow cytometric analysis, expression of LAG-3, PD-1, and TIM-3 on day 12 after transduction. Error bars represent ± SD. Data represent triplicate experiments. *, *P* < 0.05; **, *P* < 0.01; ***, *P* < 0.001, respectively by one-way ANOVA. **E,***In vitro*^51^Cr release assay of GD2-2840z-CARTs, GD2-28z-CARTs, and GD2-BBz-CARTs (effectors) against GD2^+^ tumor cells (NK-YS). Error bars represent ± SD. Data represent triplicate experiments. **F,***In vitro*^51^Cr release assay of GD2-2840z-CARTs against GD2^+^ SCLC. Error bars represent ± SD. Data represent three independent triplicate experiments. **G,** Flow cytometric analysis, MFI of GD2 on GD2-expressing tumors. Error bars represent ± SD. The bars represent triplicate experiments. **H,***In vitro*^51^Cr release assay of GD2-2840z-CARTs against SCLC cell lines. Error bars represent ± SD. Data represent triplicate experiments.

Percentages of GD2 antigen expression on the non–small cell lung cancer cell line A549 and on the four SCLC cell lines H82, H69, H446, and SCLC-J1 were 6.34%, 59.2%, 99.9%, 93.5%, and 98.4% respectively (Fig. 1A). We generated three types of lentiviral plasmid encoding a CAR-directed against GD2 antigen. The constructs comprise scFv derived from the 14g2a antibody (24) linked to sequences encoding a costimulatory domain (CD28-OX40, CD28, and 4-1BB) and CD3ζ domains (Fig. 1B). mCherry is linked to GD2-CAR via 2A-derived nucleotides, serving as a surrogate marker for CAR transgene expression.

PBMCs from a healthy donor were stimulated by anti-CD3 and -CD28 antibodies 3 days before CAR transduction with the lentiviral GD2-CAR vectors. The generated GD2-CARTs were named GD2-2840z-CARTs, GD2-28z-CARTs, and GD2-BBz-CARTs after their costimulatory domains (Fig. 1B). CAR transgene expression, measured by mCherry expression using flow cytometry, was 85.4% on GD2-2840z-CARTs, 57.4% on GD2-28z-CARTs, and 80.2% on GD2-BBz-CARTs (Fig. 1C). We compared expression of the exhaustion markers LAG-3, PD-1, and TIM-3 on GD2-2840z-CARTs, GD2-28z-CARTs, and GD2-BBz-CARTs by flow cytometry 12 days after GD2-CAR transduction. The expression of LAG-3 on GD2-2840z-CARTs was 9.4% ± 0.45% on day 12, whereas that on GD2-28z-CARTs was 16% ± 0.95% (2840z vs. 28z, *P* = 0.0032) and that on GD2-BBz-CARTs was 17.8% ± 2.23% (2840z vs. BBz, *P* = 0.0009; Fig. 1D, left). The expression of PD-1 was 6.03% ± 0.69% on GD2-2840z-CARTs, 7.14% ± 0.61% on GD2-28z-CARTs (2840z vs. 28z, *P* = 0.4376) and 10.00% ± 1.52% on GD2-BBz-CARTs (2840z vs. BBz, *P* = 0.0078; Fig. 1D, center). The expression of TIM-3 (which is not only an exhaustion marker but also an activation marker) was 54.8% ± 0.62% on GD2-2840z-CARTs, 67.33% ± 1.19% on GD2-28z-CARTs (2840z *vs*. 28z, *P* = 0.0001), and 59.10% ± 2.17% on GD2-BBz-CARTs (2840z vs. BBz, *P* = 0.0273; Fig. 1D, right). Expression of exhaustion markers on GD2-2840z-CARTs thus was the lowest among these three GD2-CAR constructs.

### GD2-2840z-CARTs Showed Cytotoxicity Against GD2-expressing Tumors, SCLC Excepted

To compare cytotoxicities of GD2-2840z-CARTs, GD2-28z-CARTs, and GD2-BBz-CARTs against GD2-expressing lymphoma cells (ref. 41; NK-YS cells), we performed ^51^Cr release assays. At an E:T ratio of 20:1, GD2-2840z-CARTs more strongly showed cytotoxicity against GD2-expressing lymphoma cells (26.2% ± 1.3%) than did GD2-28z-CARTs (10.4% ± 1.4%, *P* < 0.0001) and GD2-BBz-CARTs (19.8% ± 1.4%, *P* = 0.0007; Fig. 1E). Furthermore, we also confirmed that GD2-2840z-CARTs showed stronger cytotoxicity against T98G cells (47.9% ± 4.7%) than did GD2-28z-CARTs (34.5% ± 4.3%, *P* = 0.0063) and GD2-BBz-CARTs (19.0% ± 1.5%, *P* < 0.0001) at an E:T ratio of 40:1 (Supplementary Fig. S1A). As cytotoxic activity of GD2-2840z-CARTs was more effective than activities of GD2-28z-CARTs and GD2-BBz-CARTs, we used the GD2-2840z-CAR construct in subsequent experiments.

Cytotoxicity of GD2-2840z-CARTs against three SCLC cell lines with > 90% GD2 expression (H69; 99.9%, H446; 93.5%, SCLC-J1; 98.4%; Fig. 1A) was very low even at an E:T ratio of 40:1 [respectively 6.3% ± 0.5% (7.0% ± 1.1% control T cells), 3.2% ± 2.5% (1.7% ± 2.2% control T cells), and 11.3% ± 1.4% (8.2% ± 0.8% control T cells)] (Fig. 1F). Mean fluorescence intensity (MFI) levels of GD2 expression on six GD2-expressing tumor cell lines, including three SCLC cell lines, assessed by flow cytometry varied widely (1,503 ± 12.5 to 41,694 ± 1596.5; Fig. 1G). As the MFI level of H446 cells decreased with long-term culture, we needed to sort GD2-positive cells; accordingly, average data were utilized. At an E:T ratio of 20:1, the cytotoxicity of GD2-2840z-CARTs against T98G cells, the cell line that expressed GD2 most strongly, was 63.2% ± 0.2%; this was the cell line of the six against which cytotoxicity was strongest. SCLC-J1 cells also expressed GD2 strongly, but GD2-2840z-CARTs affected only 13.4% ± 3.0% specific lysis (Fig. 1H). On the other hand, we did not observe baseline lytic activity of control T cells against GD2-positive cells (Fig. 1F; Supplementary Fig. S1B). Using these six cell lines, we found no clear relationship between killing activity of GD2-2840z-CARTs and GD2 expression levels (Fig. 1G and H).

These results established that GD2-2840z-CARTs showed killing activity against GD2-expressing tumor cells without SCLC cells and that cytotoxicities of GD2-2840z-CARTs against these six cell lines did not directly correlate with GD2 expression levels.

### GD2-CARrejTs Exhibited Potent Cytotoxicity Against SCLC *In Vitro*

**FIGURE 2:**
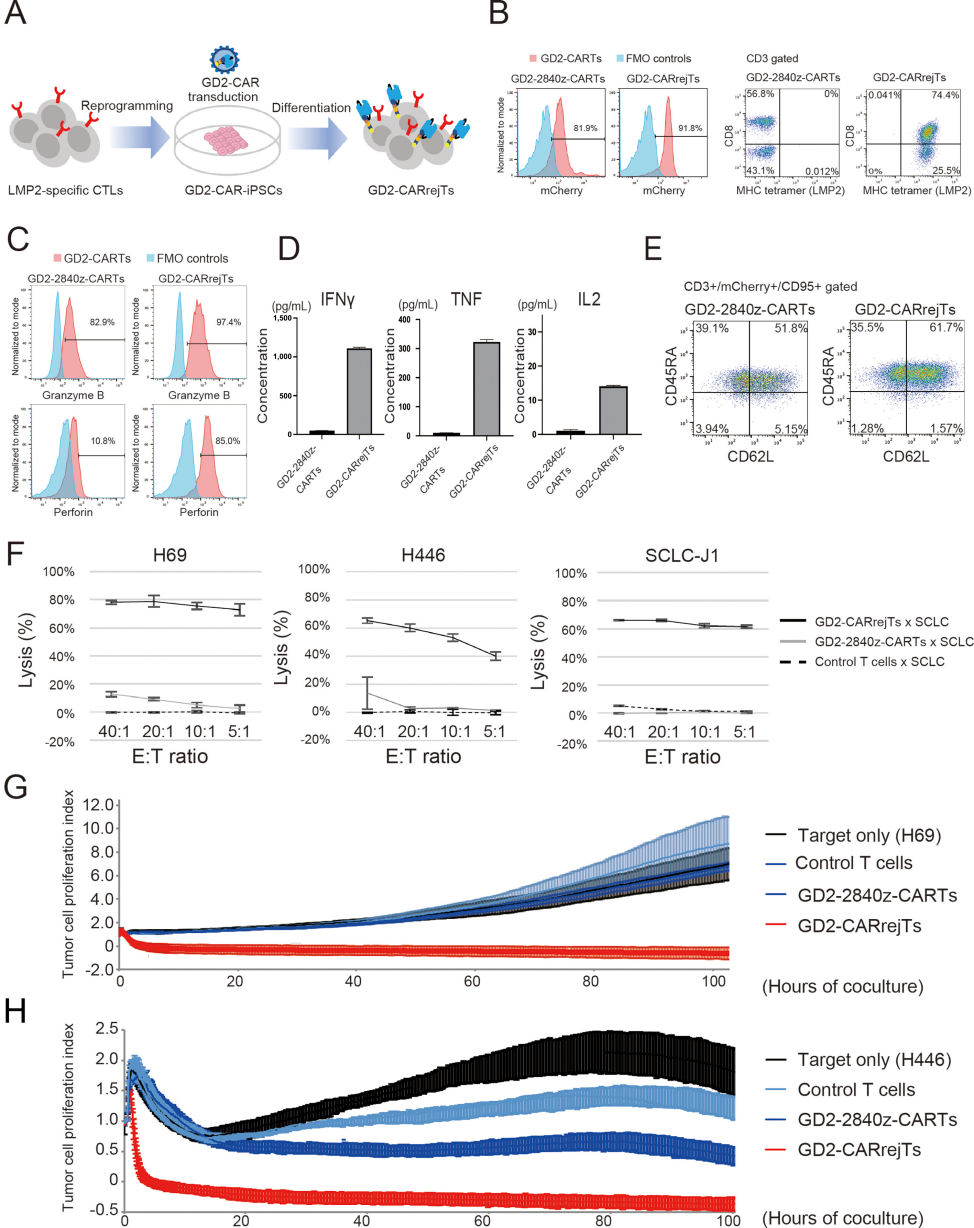
Generation of GD2-CARrejTs. **A,** Schema, generation of GD2-CARrejTs. T-iPSCs established from LMP2-specific CTL clones were transduced with lentiviral GD2-CAR and GD2-CAR-iPSCs were differentiated into GD2-CARrejTs. **B,** Flow cytometric analysis of GD2-2840z-CARTs and GD2-CARrejTs to evaluate CAR transgene expression and LMP2 antigen specificity. Histogram in blue, fluorescence minus one (FMO) control. Histogram in red, GD2-2840z-CARTs and GD2-CARrejTs. The plots represent three independent experiments. **C,** Intracellular perforin and granzyme B marking, GD2-2840z-CARTs and GD2-CARrejTs (red). Histogram in blue, FMO control. The plots represent three independent experiments. **D,** CBA for measuring cytokines (IFNγ, TNF, and IL2) produced by effector T cells (GD2-2840z-CARTs and GD2-CARrejTs) after 24 hours of coculture with SCLC-J1. Error bars represent ± SEM. **E,** Representative flow cytometrogram of memory phenotype (CD45RA and CD62 L population) among GD2-2840-CARTs and GD2-CARrejTs. **F,***In vitro*^51^Cr assays of GD2-2840z-CARTs, GD2-CARrejTs, and control T cells against SCLC cell lines. Error bars represent ± SD. Data represent three independent triplicate experiments. **G,** RTCA continuous graphical output of tumor proliferation indices up to 40 hours for H69 cells cocultured with control T cells, GD2-2840z-CARTs, and GD2-CARrejTs and for tumor only; E:T ratio uniformly 1:1. Data were plotted and are shown as mean ±SD. Data represent three independent triplicate experiments. **H,** RTCA continuous graphical output of tumor proliferation indices up to 100 hours for H446 cells cocultured with control T cells, GD2-2840z-CARTs, and GD2-CARrejTs and for tumor only; E:T ratio uniformly 1:1. Data were plotted and are shown as mean ±SD. Data represent three independent triplicate experiments.

We generated iPSC-derived GD2-CARTs by introducing GD2-CAR into LMP2-rejTs (GD2-CARrejTs). An LMP2-CTL clone (TYGPVFMSL) generated from a healthy donor (29) was reprogrammed into iPSCs with the Sendai virus vector encoding six reprogramming factors (*OCT3/4*, *SOX2*, *KLF4*, *c-MYC*, *NANOG*, and *LIN28*; ref. 28). The iPSCs were transduced with lentiviral GD2-2840z-CAR vector (Fig. 1B). To prevent silencing of the GD2-CAR transgene, transduced iPSCs were sorted three times for mCherry expression before GD2-CAR-iPSCs were differentiated into GD2-CARrejTs (Fig. 2A). CAR transgene expression by GD2-2840z-CARTs and GD2-CARrejTs was evaluated by flow cytometry for mCherry expression, with respectively 81.9% and 91.8% CAR transgene expression found (Fig. 2B). Flow cytometry determined LMP2 (TYGPVFMSL) antigen specificity of GD2-2840z-CARTs and GD2-CARrejTs as respectively 0% and 99.9% (74.4% CD8^+^CD4^−^, 25.5% CD8^−^CD4^−^; Fig. 2B). To measure the cytotoxic potential of GD2-2840z-CARTs and GD2-CARrejTs, intracellular staining for granzyme B and perforin was performed on both cells in their unstimulated state. Both GD2-2840z-CARTs and GD2-CARrejTs demonstrated marked cytotoxic potential, with high expression of granzyme B (82.9% and 97.4%, respectively). However, expression of perforin was distinctly lower in GD2-2840z-CARTs than in GD2-CARrejTs (10.8% vs. 85.0%; Fig. 2C). Furthermore, we measured cytokine release levels (IFNγ, TNF, IL2, IL4, IL6, IL10, and IL17A) of GD2-2840z-CARTs and GD2-CARrejTs cocultured with SCLC-J1 cells overnight. GD2-CARrejTs displayed significantly higher levels of cytokine release (IFNγ, TNF, IL2, IL4, and IL10) than did GD2-2840z-CARTs (IFNγ, TNF, IL2, IL4, and IL10; *P* < 0.0001, IL6, IL17A; ns; Fig. 2D; Supplementary Fig. S2A). The memory phenotypes of GD2-2840z-CARTs and GD2-CARrejTs were also evaluated by flow cytometry; the proportion of stem cell memory phenotype (CD3^+^, CD45RA^+^, CD62L^+^, CD95^+^) was 51.8% in GD2-2840z-CARTs and 61.7% in GD2-CARrejTs (Fig. 2E; Supplementary Fig. S2B). To investigate whether GD2-CARrejTs more strongly exhibited cytotoxicity against SCLC than did GD2-CARTs, 51Cr release assays were performed. Cytotoxicity of GD2-CARrejTs was overwhelmingly stronger against H69, H446, and SCLC-J1 cells than was that of GD2-2840z-CARTs (H69; 78.2% vs. −0.4%; *P* < 0.0001, H446; 65.2% vs. −0.2%; *P* = 0.0015, SCLC-J1; 66.1% vs. −0.2%; *P* < 0.0001) at an E:T ratio of 40:1 (Fig. 2F).

We further investigated whether the low percentage of CD8 positivity in GD2-2840z-CARTs influences their cytotoxicity against SCLC cells. ^51^Cr release assays using CD8-positive sorted GD2-2840z-CARTs were performed (Supplementary Fig. S2C and S2D). Although CD8 sorted GD2-2840z-CARTs exhibited slightly greater cytotoxicity against H446 cells than did GD2-2840z-CARTs (without CD8 sort), cytotoxicity against H69 and SCLC-J1 cells was not significantly enhanced (H69; 21.2% vs. 13.6%; *P* = 0.2326, H446; 21.2% vs. 13.3%; *P* = 0.0029, SCLC-J1; 5.6% vs. 5.2%; *P* = 0.9919 at an E:T ratio of 20:1; Supplementary Fig. S2D).

We also compared the cytotoxicity against H69 cells of GD2-2840z-CARTs with that of GD2-CARrejTs over a long period of time using RTCA. H69 cells were cocultured with control T cells, GD2-2840z-CARTs, and GD2-CARrejTs. Although H69 cells continuously proliferated in the absence of T cells, this was immediately suppressed on coculture with GD2-CARrejTs (1 hour; 7.3% ± 2.6% killing, 2 hours, 71.3% ± 10.0% killing). GD2-CARrejTs completely suppressed tumor growth for more than 80 hours. The cytotoxicity of GD2-CARrejTs against H69 cells was 93.4% ± 7.8% after 80 hours of coculture with H69 cells, whereas the cytotoxicities of control T cells and GD2-2840z-CARTs were only 0.9% ± 4.5% and 2.8% ± 6.5%, respectively (GD2-CARrejTs vs. control T cells, GD2-CARrejTs *vs.* GD2-2840z-CARTs; both *P* < 0.0001; Fig. 2G). Cytotoxicity against H446 cells was similarly examined. GD2-CARrejTs also robustly suppressed tumor growth from the onset of coculture, without reattachment of H446 cells during the observation period (80 hours; 99.0% ± 3.2%). GD2-2840z-CARTs evinced scarcely any cytotoxicity against H446 cells at the onset of coculture, but as time passed cytotoxicity appeared (80 hours; 61.1% ± 4.6%). However, the cytotoxicity of GD2-CARrejTs against H446 cells was significantly higher than that of GD2-2840z-CARTs throughout the study (10 hours, *P* < 0.0001; 80 hours, *P* < 0.0001; Fig. 2H).

We concluded that GD2-CARrejTs expressing high levels of granzyme B and perforin and releasing large amounts of IFNγ, TNF, and IL2 rapidly exerted more robust cytotoxicity against SCLC than did GD2-2840z-CARTs. Furthermore, the cytotoxicity of GD2-CARrejTs was sustained over time, while GD2-2840z-CARTs, which were generated using the same CAR construct, did not exhibit the high levels of SCLC tumor suppression against SCLC that GD2-CARrejTs showed.

### Robust Anti-SCLC Effect and Survival Advantage of GD2-CARrejTs *In Vivo*

**FIGURE 3:**
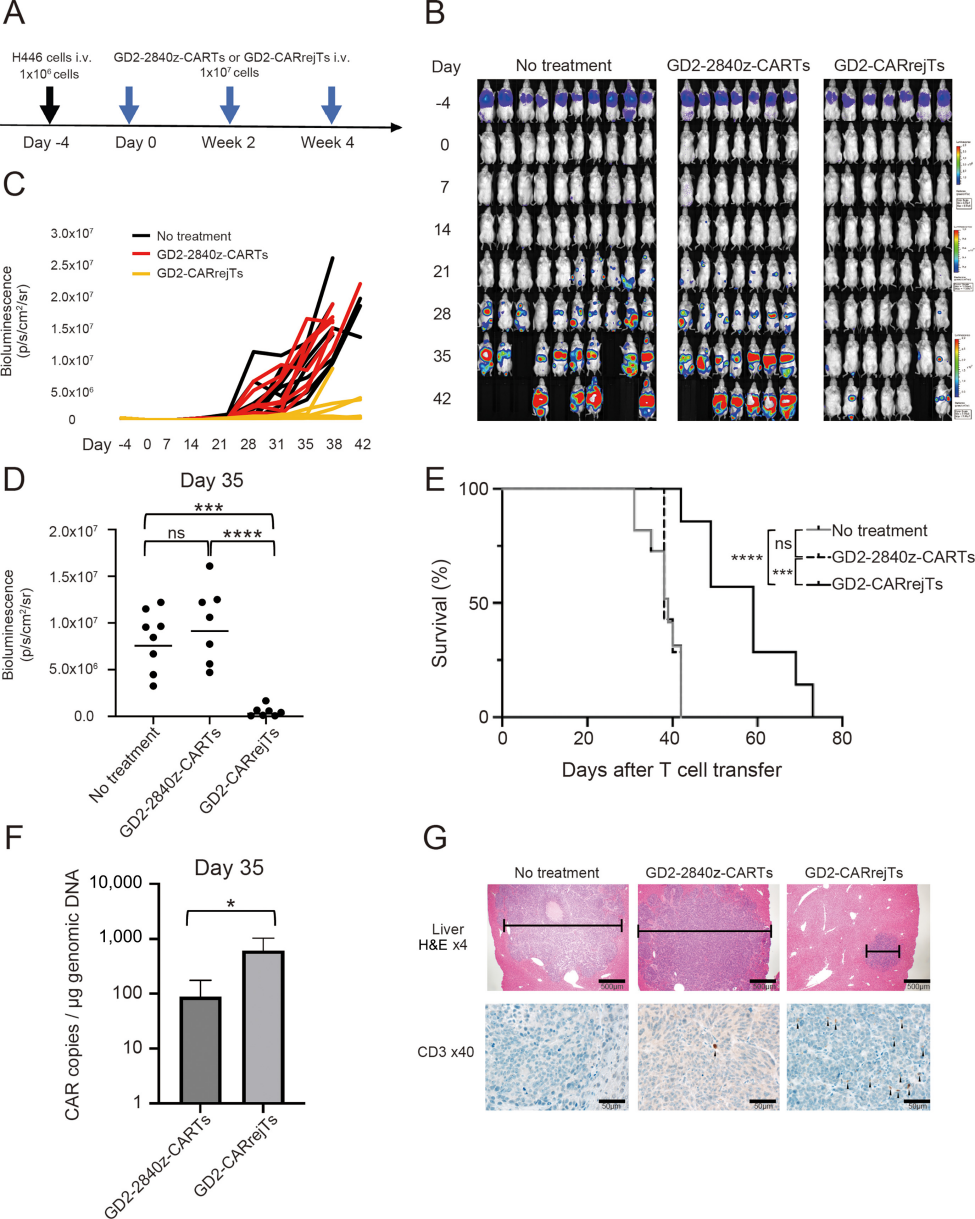
Robust tumor suppressive effect of GD2-CARrejTs *in vivo.***A,** Graphical abstract of *in vivo* experiment. H446 cells labeled with FFluc/GFP were intravenously injected into NOG mice (1 × 10^6^ cells/mouse) 4 days before treatment. Mice treated with GD2-2840z-CARTs and GD2-CARrejTs were treated every 2 weeks (three doses). **B,** Bioluminescence imaging of mice engrafted with H446 cells in three groups: no treatment group (control group, *n* = 10), GD2-2840z-CARTs group (*n* = 7), and GD2-CARrejTs group (*n* = 7). Images of all mice from each group of three independent experiments are shown. **C,** Tumor burden of each mouse is shown, comparing the *in vivo* efficacy of GD2-2840z-CARTs and GD2-CARrejTs. Bioluminescence kinetics of all mice from each group of three independent experiments are shown: no treatment group (control group, *n* = 10), GD2-2840z-CARTs group (*n* = 7), and GD2-CARrejTs group (*n* = 7). **D,** Quantitation of tumor burden on day 35 is shown: no treatment group (*n* = 8), GD2-2840z-CARTs group (*n* = 7), and GD2-CARrejTs group (*n* = 7). ****, *P* < 0.0001 by one-way ANOVA. **E,** Kaplan–Meier curves representing percentage survival of groups: no treatment group (*n* = 10), GD2-2840z-CARTs group (*n* = 7), and GD2-CARrejTs group (*n* = 7). ***, *P* < 0.001; ****, *P* < 0.0001 by log-rank test. ns, not significant. **F,** Copy numbers of GD2-CAR transduced T cells in mouse treated with GD2-2840z-CARTs and GD2-CARrejTs on day 35. Error bars represent ± SD. The plots represent three independent experiments. **G,** Photomicrographs, representative hematoxylin and eosin–stained sections of liver of SCLC-bearing mouse in each group; no treatment, GD2-2840z-CART treatment, and GD2-CARrejT treatment (top row). Infiltrates are bracketed. Human CD3^+^ T-cell infiltrates in liver (bottom row). CD3^+^ T cells are stained in brown (black arrowhead). 4x, 40x: Objective lens. Scale bars, each 20 µm.

To observe the antitumor effect of GD2-CARrejTs against SCLC *in vivo*, H446 cells labeled with FFluc/GFP were intravenously injected into NOG mice (1 × 10^6^ cells/mouse). Four days after FFluc-GFP-H446 injection, mice were divided into a control group (no treatment; *n* = 10) and two treatment groups: GD2-2840z-CARTs (*n* = 7) and GD2-CARrejTs (*n* = 7; Fig. 3A). Bioluminescence rapidly increased on day 21 in the untreated group and the GD2-2840z-CART treatment group (Fig. 3B and C). Tumor signal was clearly suppressed in mice treated with GD2-CARrejTs (Fig. 3B and C). No significant difference in tumor signal was observed between untreated mice and GD2-CART treated mice 35 days after the first treatment (*P* = 0.3840, one-way ANOVA; Fig. 3D). In contrast, the tumor signal in mice treated with GD2-CARrejTs was significantly lower than in mice treated with GD2-2840z-CARTs on day 35 (no treatment vs. GD2-CARrejTs; *P* = 0.0003, GD2-2840z-CARTs vs. GD2-CARrejTs, *P* <0.0001; Fig. 3D). Mice treated with GD2-CARrejTs survived significantly longer (42–73 days) than untreated mice (31–42 days, *P* = 0.0006) and GD2-2840z-CART treated mice (38–42 days, *P* = 0.0003), and mice treated with GD2-2840z-CARTs did not survive longer than untreated mice (*P* = 0.7179; Fig. 3E). To evaluate persistence of GD2-2840z-CARTs and GD2-CARrejTs *in vivo*, quantitative real-time PCR was performed. On day 35, GD2-CAR copy numbers in peripheral blood differed significantly between mice treated with GD2-2840z-CARTs and mice treated with GD2-CARrejTs (*P* = 0.0487, unpaired *t* test; Fig. 3F).

### Dense Infiltration of Tumor by GD2-CARrejTs Demonstrated *In Vivo*

To learn whether GD2-2840z-CARTs and GD2-CARrejTs infiltrated tumors, we on day 35 euthanized and dissected one mouse from each group, with sampling for histopathologic examination. Multiple tumors in the liver and kidney were observed in both the untreated mouse and the mouse treated with GD2-2840z-CARTs, while no tumors were observed in the lungs of these mice. Only a single small tumor was observed in the liver of the mouse treated with GD2-CARrejTs, and the lungs, kidneys, spleen, and intestine were macroscopically tumor-free. Although human CD3 T^+^ cells were rare within liver tumors in the mouse treated with GD2-2840z-CARTs, human CD3 T^+^ cells substantially infiltrated intratumoral vessels in the liver of the mouse treated with GD2-CARrejTs. This demonstrated that GD2-CARrejTs effectively infiltrated and survived in tumor *in vivo*. We infer that they continuously inhibited tumor growth. By far most GD2-2840z-CARTs, however, did not infiltrate tumor (Fig. 3G). The clear difference in the number of human CD3^+^ cells infiltrating tumors in 2 mice, one treated with GD2-2840z-CARTs and one with GD2-CARrejTs, suggested that GD2-CARrejTs more robustly persist *in vivo* than do GD2-2840z-CARTs and that they more efficiently seek out tumor. The superior ability of GD2-CARrejTs to accumulate in tumors compared with GD2-2840z-CARTs on day 35 is thought potentially to contribute to the clear extension of survival periods observed in the group treated with GD2-CARrejTs.

From these results, we concluded that GD2-CARrejTs with the strong ability to accumulate in tumors exhibited a robust tumor suppressive effect against SCLC *in vivo* and conferred a clear survival advantage.

### Low Levels of Gene Expression with TIGIT in GD2-CARrejTs by scRNA-seq

**FIGURE 4:**
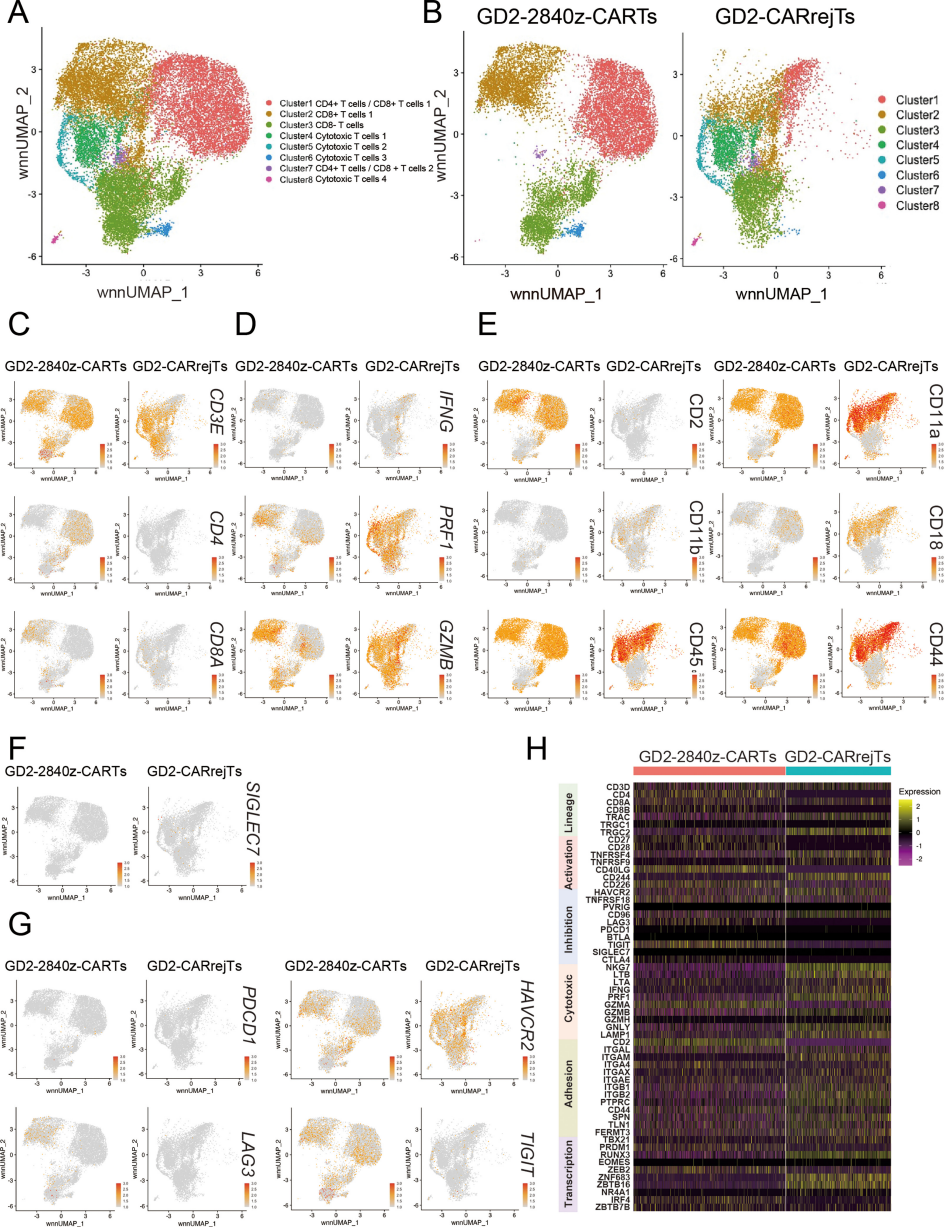
scRNA-seq analysis of GD2-2840-CARTs and GD2-CARrejT. **A** and **B,** UMAP projection of scRNA-seq data of GD2-2840z-CARTs and GD2-CARrejTs. Cluster gene characteristics determine cluster functional description. Cluster 1: CD4^+^ T cells/CD8^+^ T cells 1; Cluster 2: CD8^+^ T cells 1; Cluster 3: CD8^−^ T cells; Cluster 4: Cytotoxic T cells 1; Cluster 5: Cytotoxic T cells 2; Cluster 6: Cytotoxic T cells 3; Cluster 7: CD4^+^ T cells/CD8^+^ T cells 2; Cluster 8: Cytotoxic T cells 4. Cells colored according to single-cell genotype group. **C,** Lineage (*CD3E*, *CD4*, *CD8A*). **D,** Cytotoxicity (*IFNG*, *PRF1*, *GZMB*). **E,** Immunologic synapse (CD2, CD11a, CD11b, CD45, CD44). **F,** Inhibitory immunoreceptor (*SIGLEC7*). **G,** Exhaustion (*HAVCR2*, *LAG-3*, *TIGIT*, *PD-1*). **H,** Heat map of unique signature genes (lineage, costimulation, coinhibition, cytotoxic molecules, transcription factors) for each individual cluster.

To determine contributors to the marked discrepancy in cytotoxicity against SCLC between GD2-2840z-CARTs and GD2-CARrejTs, we performed scRNA-seq and CITE-seq. UMAP visualization revealed eight individual clusters (Fig. 4A). UMAP plots for each sample were also displayed separately to distinguish cellular distribution patterns unique to GD2-2840z-CARTs and GD2-CARrejTs (Fig. 4B). Furthermore, expression patterns of genes and antigens on UMAP (Fig. 4C–G) and heat map (Fig. 4H) were shown. CD8 T cells were mainly found in clusters 2, 4, and 5 (Fig. 4A–C), which expressed genes associated with cytotoxicity (*IFNG, PRF1,* and *GZMB*) at higher levels in GD2-CARrejTs than in GD2-2840z-CARTs (Fig. 4D). GD2-CARrejTs expressed proteins associated with adhesion molecules and immunologic synapse at high levels (CD11a, CD11b, CD18, CD44, and CD45; Fig. 4E). CD11a (*ITGAL*) and CD18 (*ITGB2*), which make up lymphocyte function-associated antigen 1 (LFA-1), in particular were more highly expressed in GD2-CARrejTs than in GD2-2840z-CARTs (Fig. 4E and H). The inhibitory immunoreceptor *SIGLEC7*, a ligand of GD2 (42), was not found on either GD2-2840z-CARTs or GD2-CARrejTs (Fig. 4F and H). By contrast, GD2-2840z-CARTs expressed gene encoding inhibitory immunoreceptor *CTLA4* at a higher level than did GD2-CARrejTs (Fig. 4H). Because GD2-CAR reportedly induces a tonic signal that rapidly exhausts T cells (43), we assessed exhaustion in GD2-2840z-CARTs and GD2-CARrejTs. Expression levels of exhaustion markers such as PD-1 (*PDCD1)* and TIM3 (*HAVCR2*), which also serves as a marker of CART cell activation (44), were similar in GD2-2840z-CARTs and GD2-CARrejTs. On the other hand, expression of *LAG3* and *TIGIT* was significantly lower (both *P* < 0.0001) in GD2-CARrejTs than in GD2-2840z-CARTs (Fig. 4G and H). We compared expression of genes encoding activation markers, inhibitory markers, and transcription factors. Gene expression associated with activation markers such as *TNFRSF4*, *TNFRSF9*, and *CD244* was high in GD2-CARrejTs (Fig. 4H). Although *CD40LG* gene expression was low in GD2-CARrejTs, protein expression levels (CD154) were similar in GD2-CARrejTs and GD2-2840z-CARTs. In addition, expression of genes encoding transcription factors known to be cytotoxic effectors (*TBX21* and *RUNX3*) and to mark tissue resident memory T cells (*ZNF683* and *ZBTB16*) were high in GD2-CARrejTs (Fig. 4H). We also compared expression levels of these genes in GD2-2840z-CARTs, GD2-CARrejTs, and healthy donor-derived CD3^+^ T cells as control T cells. Control T cells expressed genes associated with exhaustion markers (such as TIGIT and LAG3) and activation marker CD226 at low levels (Supplementary Fig. S3A).

In addition to scRNA-seq, CITE-seq analyses revealed that GD2-CARrejTs expressed CD8αα^+^, CD4^−^, CD56^+^, CD2^−^, CD5^−^, CD27^low^, CD28^+^, CD154^+^, and CD226^+^, whereas GD2-CARTs expressed CD8αβ^+^/CD4^+^, CD56^−^, CD2^+^, CD5^+^, CD27^low^, CD28^+^, CD154^+^, and CD226^+^ (Fig. 4; Supplementary Fig. S3B and S3C). Furthermore, gene expression associated with chemokine receptors such as *CCL1* (45), *CCR1*, *CCR6* (46), *CXCR3* (47), *XCL1* (48), and *XCL2* (49) was at higher levels in GD2-CARrejTs than in GD2-CARTs (Supplementary Fig. S3D). This may also contribute to the robust antitumor effect of GD2-CARrejTs against SCLC.

These scRNA-seq analyses indicated that GD2-CARrejTs more strongly expressed genes associated with cytotoxicity, the immunologic synapse, and transcription factors than did GD2-2840z-CARTs. Furthermore, expression levels of genes associated with T-cell exhaustion, especially *TIGIT* and *LAG3*, were clearly lower in GD2-CARrejTs than in GD2-2840z-CARTs.

### Blockade of TIGIT and PD-1 Augments Cytotoxicity of GD2-2840z-CARTs

**FIGURE 5:**
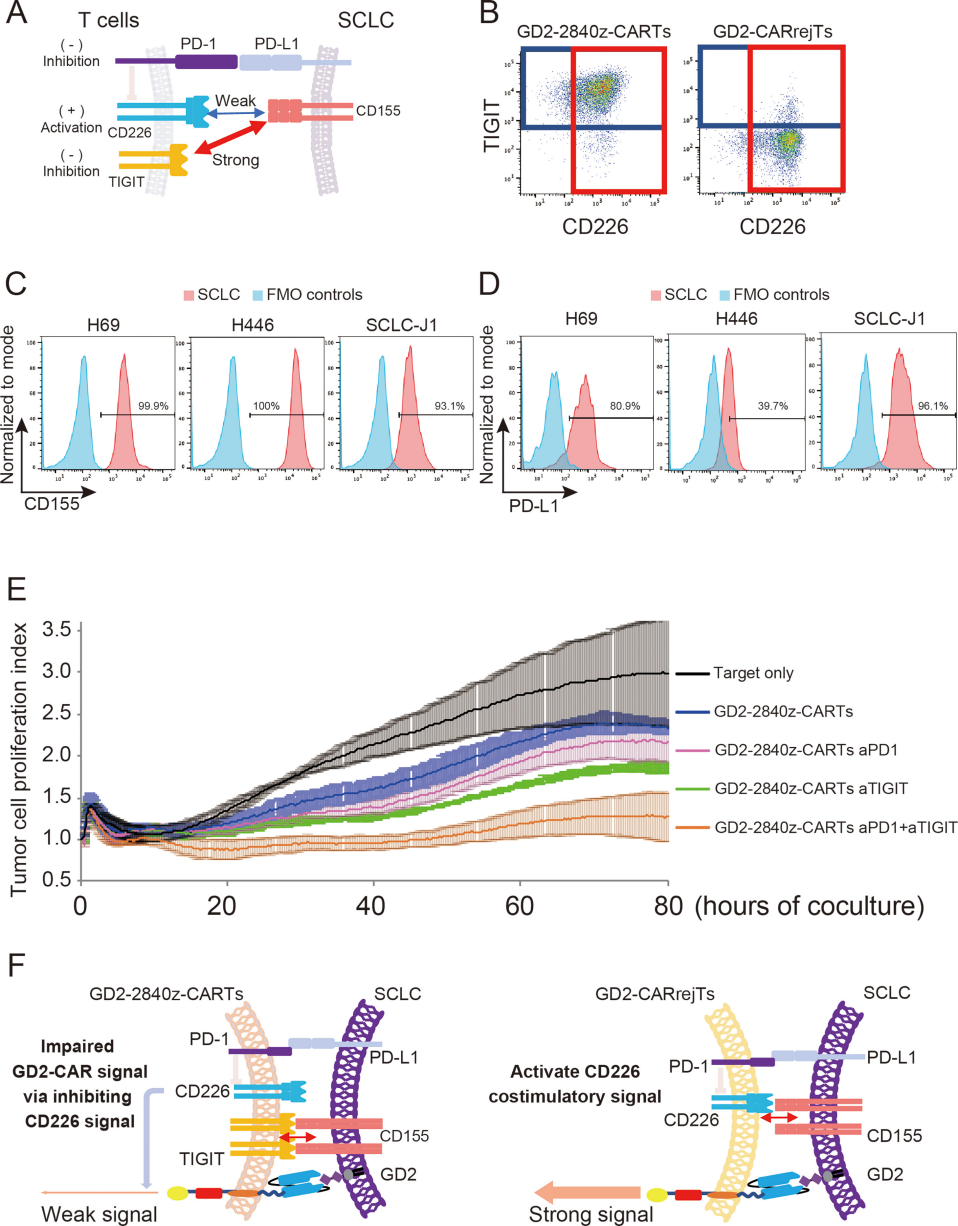
Blockade of TIGIT and PD-1 augments cytotoxicity of GD2 2840z-CARTs. **A,** Schema of immune-regulatory mechanism of T cells against SCLC. **B,** Flow cytometric analysis of TIGIT and CD226 expression on GD2-2840z-CARTs and GD2-CARrejTs. The plots represent independent triplicate experiments. **C,** Flow cytometric analysis of CD155 expression on H69, H446, and SCLC-J1 (red). Histogram in blue, fluorescence minus one (FMO) control. The histograms represent independent triplicate experiments. **D,** Flow cytometric analysis of PD-L1 expression on GD2-expressing tumor cell lines (H69, H446, and SCLC-J1; red). Histogram in blue, FMO control. The histograms represent independent triplicate experiments. **E,** RTCA continuous graphical output of tumor proliferation indices up to 80 hours alone and in coculture with GD2-2840z-CARTs with/without anti-TIGIT (aTIGIT) and/or anti-PD-1 antibodies (aPD-1). Data were plotted and are shown as mean ± SD. Data represent three independent triplicate experiments. **F,** Schema of immune-regulatory mechanism of GD2-2840z-CARTs and GD2-CARrejTs against SCLC.

scRNA-seq and CITE-seq results led us to hypothesize that high expression of *TIGIT* on GD2-2840z-CARTs may contribute to low cytotoxicity of GD2-2840z-CARTs against SCLC. TIGIT acts as an inhibitory receptor by binding strongly to CD155 instead of CD226 (ref. 50; Fig. 5A). To explore the effect of TIGIT expression on T-cell activity of GD2-CARTs, we first examined TIGIT and CD226 expression levels on GD2-2840z-CARTs and GD2-CARrejTs by flow cytometry. While 94.2% of GD2-2840z-CARTs expressed TIGIT, only 6.0% of GD2-CARrejTs did so. The costimulatory counterpart to TIGIT, CD226, was highly expressed on GD2-CARTs (90.5%) and GD2-CARrejTs (91.6%; Fig. 5B). We also confirmed that positivity for PD-1, TIGIT, and CD226 in GD2-2840z-CARTs and GD2-CARrejTs was not clearly altered after 24 hours of coculturing with SCLC-J1 cells (Supplementary Fig. S4A). CD155 expression on SCLC cells was > 90% in all cell lines examined (H69, 99.9%; H446, 100%; SCLC-J1, 93.1%; Fig. 5C).

Figure 5A illustrates how the costimulatory pathway induced by activation of CD226 was impaired not only by TIGIT but also by PD-1 which binds to PD-L1 (51). All SCLC cell lines we used in this study also expressed PD-L1 (H69, 80.9%; H446, 39.7%; SCLC-J1, 96.1%; Fig. 5D).

To see whether anti-TIGIT antibody could restore effector function of GD2-2840z-CARTs against SCLC, we used RTCA to compare antitumor activity of GD2-2840z-CARTs with or without anti-TIGIT antibody against H446 cells. As expected, GD2-2840z-CART cytotoxicity against H446 cells did not differ, whether cocultured with or without anti-TIGIT antibody, even after 80 hours (36.2% ± 1.7% and 21.0% ± 2.5% respectively, *P* = 0.1428, one-way ANOVA; Fig. 5E). On the other hand, coblockade of TIGIT and PD-1 augmented the cytotoxicity of GD2-2840z-CARTs against H446 cells. Ten hours after starting coculture, GD2-2840z-CARTs treated with anti-PD-1 and anti-TIGIT antibodies exhibited no significant increase in cytotoxicity over that of GD2-2840z-CARTs alone. However, the cytotoxicity against H446 cells of GD2-2840z-CARTs with anti-PD-1 and anti-TIGIT antibodies gradually increased (20 hours, 32.6% ± 7.7%), finally yielding a significant increase over that of GD2-2840z-CARTs alone (80 hours, 54.9% ± 9.2% and 21.0% ± 2.5%, *P* = 0.0012, one-way ANOVA; Fig. 5E). On the other hand, the cytotoxicity against H446 cells of both GD2-CARrejTs and control T cells with anti-PD-1 and anti-TIGIT antibodies did not significantly increase (Supplementary Fig. S4B).

With cytotoxic function of GD2-2840z-CARTs enhanced by coblockade of PD-1 and TIGIT (Fig. 5E), even GD2-CARrejTs with low expression of TIGIT and PD-1 acquired robust antitumor effect against SCLC (Fig. 5F).

## Discussion

Constitutive phosphorylation of the GD2-CAR CD3-ζ domain due to CAR clustering in an antigen-independent manner reportedly leads to early exhaustion of CART cells, an obstacle to their clinical deployment (43). In this study, GD2-CARTs expressed exhaustion markers (LAG-3, PD-1, and TIM-3) only 12 days after GD2-CAR transduction (Fig. 1D). Furthermore, scRNA-seq revealed that GD2-2840z-CARTs expressed genes associated with exhaustion at levels significantly higher than those of GD2-CARrejTs (Fig. 4G and H).

Because expression of *TIGIT* was clearly lower on scRNA-seq in GD2-CARrejTs than in GD2-2840z-CARTs, we focused on *TIGIT* as a potential contributor to inefficacy of GD2-2840z-CARTs against SCLC. TIGIT is an immunoglobulin superfamily member expressed on T cells and natural killer cells. TIGIT on CD8 T cells regulates costimulatory receptor CD226 through sharing with it the ligand CD155. TIGIT binds more strongly to CD155 than does CD226. Outcompeting CD155 away from CD226 attenuates effector T-cell function (Fig. 5A and F; ref. 51). In addition, PD-1–PD-L1 interaction inhibits not only the CD28 signaling pathway but also the CD226 signaling pathway (Fig. 5A; refs. 52, 53). Although single blockade of either TIGIT or PD-1 slightly improved GD2-2840z-CART cytotoxicity against SCLC, dual blockade of TIGIT and PD-1 significantly enhanced it. These results indicated that blockade of both TIGIT and PD-1 was important in restoring effector function of GD2-directed T-cell therapy against SCLC cells. Dual downregulation of TIGIT and PD-1 enhances short-term effector function through PD-1 suppression, while TIGIT downregulation is responsible for maintaining a less exhausted state (54). Therefore, GD2-CARrejTs that express both TIGIT and PD-1 at low levels can sustain cytotoxic activity with diminished susceptibility to exhaustion, a feature that holds therapeutic promise against SCLC.

Furthermore, GD2-CARrejTs with higher proportion of stem cell memory T cells (Fig. 2E), produced perforin, IFNγ, TNF, IL2, IL4, and IL10 at higher levels than did GD2-CARTs, leading to an advantage in GD2-CARrejTs against SCLC. This high cytotoxic potential of GD2-CARrejTs could be explained by differences between original cells; GD2-CARrejTs were derived from LMP2-specific CTLs, but GD2-2840z-CARTs were derived from bulk activated T cells.

scRNA-seq analysis revealed that GD2-CARrejTs expressed genes associated with cytotoxicity (*PRF1*, *GZMB*, *IFNG*, *NKG7*, *LTB*, *LTA*, *GNLY*, and *LAMP1*; Fig. 4D and H) and genes associated with the immunologic synapse at levels higher than those of GD2-2840z-CARTs (Fig. 4E and H). LFA-1 (CD11a/CD18) is activated by CD226 binding to CD155 and activated LFA-1 allows binding to ICAM-1 with high affinity, enhancing cytotoxicity by forming an immunological synapse with ICAM-1 (55). Therefore, examination of tumor infiltration markers such as LFA-1 and ICAM-1 could be important. We infer that potent cytotoxic potential and robust immunologic synapse formation in GD2-CARrejTs may contribute to effective targeting and elimination of SCLC.

The greatest advantage of iPSC-derived T-cell therapy is the unlimited supply of therapeutic T cells feasible for use “off-the-shelf”. We have developed clinical grade HLA class I-edited iPSCs that were established from CTLs to evade rejection by recipient immune cells. GD2-CAR vector transduction into these gene-edited iPSCs will simultaneously make allogeneic GD2-CARrejT therapy available for many patients with SCLC. Although SCLC is extremely difficult to treat, GD2-CARrejTs can be prepared from iPSCs in virtually unlimited quantities, allowing multiple administrations and conferring enhanced efficacy.

In conclusion, GD2-CARrejTs demonstrated robust efficacy against SCLC both *in vitro* and *in vivo*. These findings may lead to promising therapy using iPSC technology in patients with SCLC.

## Supplementary Material

Supplementary Figure 1 Supplementary Figure S1 illustrates the cytotoxic effects of GD2-CARTs (GD2-2840z-CARTs, GD2-28z-CARTs, and GD2-BBz-CARTs) and control T cells on GD2+ tumor cells through in vitro 51Cr release assays.

Supplementary Figure 2 Supplementary Figure S2 showcases the functional and phenotypic analysis of GD2-CARTs and GD2-CARrejTs, including cytokine production (IL-4, IL-6, IL-10, and IL-17A) through cytometric bead assays, flow cytometry gating strategies, memory phenotype subsets, CAR transgene expression, and CD4/8 ratios.

Supplementary Figure 3 Supplementary Figure S3 demonstrates the expression profiles of signature genes (including lineage, co-stimulation, co-inhibition, cytotoxic molecules, and transcription factors) in GD2-2840z-CARTs, GD2-CARrejTs, and control T cells through a heatmap. It also includes UMAP projections of CITE-seq data highlighting the distribution of CD3, CD4, and CD8 markers, and phenotypic markers specific to GD2-2840z-CARTs and GD2-CARrejTs. Additionally, a heatmap showcases the expression of chemokine receptor-related genes in these cell types, providing insights into their functional characteristics.

Supplementary Figure 4 Supplementary Figure S4 presents flow cytometric analysis of PD-1, TIGIT, and CD226 expression on GD2-2840z-CARTs and GD2-CARrejTs after coculture with SCLC-J1 cells, based on three independent experiments. It also features real-time cell analysis (RTCA) showing tumor proliferation indices over 80 hours in various conditions: alone, in coculture with GD2-CARrejTs, and control T cells, with and without anti-TIGIT and anti-PD-1 antibodies. The data illustrate the effect of these treatments on tumor growth.
