# Supplementary material for: Rejuvenated iPSC-derived GD2-directed CART Cells Harbor Robust Cytotoxicity Against Small Cell Lung Cancer
Source: Cancer Res Commun. 2024 Mar 11;4(3):723–37. doi: 10.1158/2767-9764.CRC-23-0259 (PMC10926899; doi:10.1158/2767-9764.CRC-23-0259)
Supplement: Supplementary Figure 1 — Supplementary Figure S1 illustrates the cytotoxic effects of GD2-CARTs (GD2-2840z-CARTs, GD2-28z-CARTs, and GD2-BBz-CARTs) and control T cells on GD2+ tumor cells through in vitro 51Cr release assays. [file crc-23-0259-s01.docx]

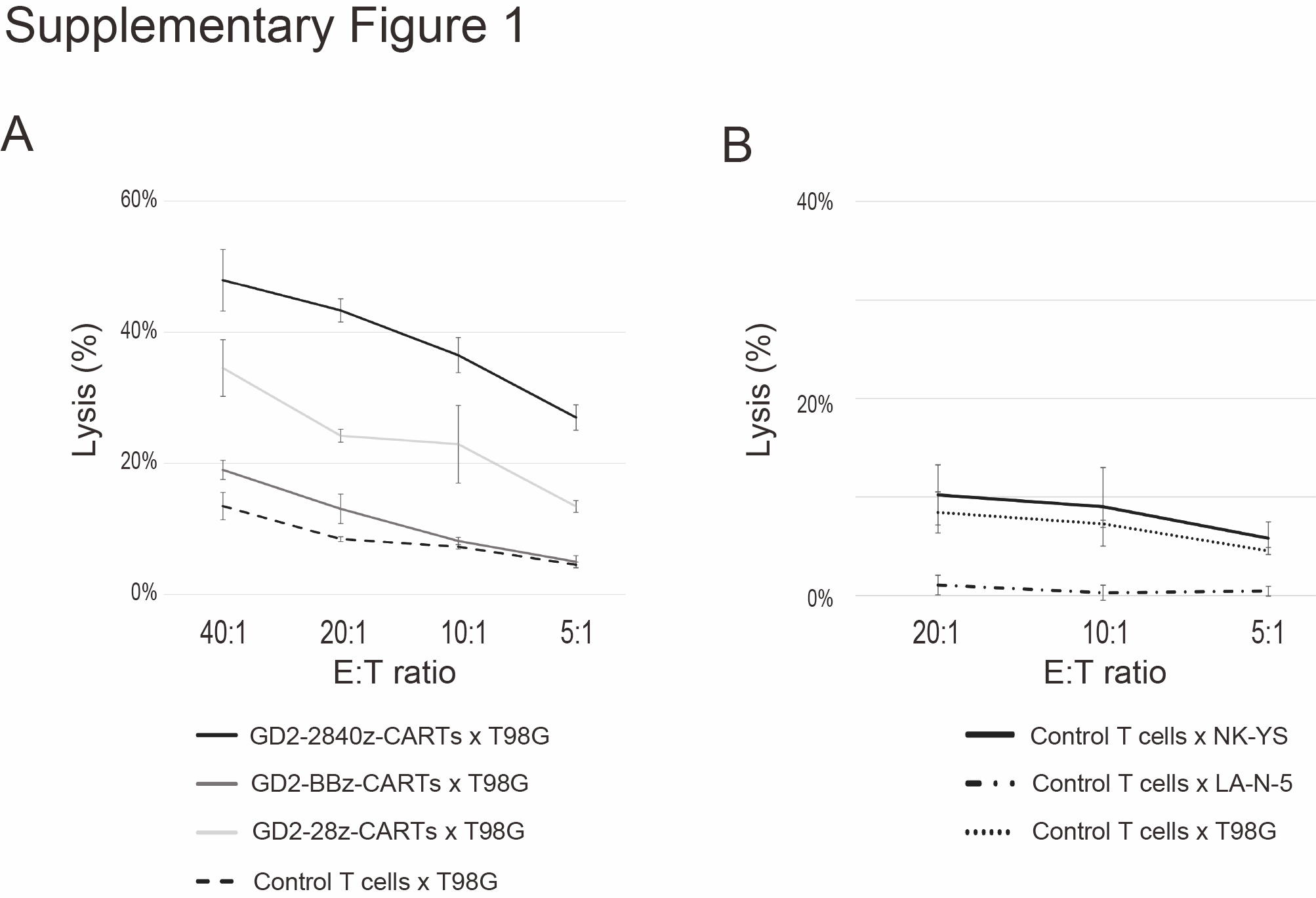


Supplementary Figure 1. Cytotoxicity of GD2-CARTs and control T cells

(A) *In vitro* ^51^Cr release assay of GD2-2840z-CARTs, GD2-28z-CARTs, and GD2-BBz-CARTs (effectors) against GD2^+^ T98G cells. Error bars represent ± SD. (B) *In vitro* ^51^Cr release assay of control T cells against GD2^+^ tumor cells (NK-YS, LA-N-5, T98G). Error bars represent ± SD.
