# Supplementary material for: Rejuvenated iPSC-derived GD2-directed CART Cells Harbor Robust Cytotoxicity Against Small Cell Lung Cancer
Source: Cancer Res Commun. 2024 Mar 11;4(3):723–37. doi: 10.1158/2767-9764.CRC-23-0259 (PMC10926899; doi:10.1158/2767-9764.CRC-23-0259)
Supplement: Supplementary Figure 2 — Supplementary Figure S2 showcases the functional and phenotypic analysis of GD2-CARTs and GD2-CARrejTs, including cytokine production (IL-4, IL-6, IL-10, and IL-17A) through cytometric bead assays, flow cytometry gating strategies, memory phenotype subsets, CAR transgene expression, and CD4/8 ratios. [file crc-23-0259-s02.docx]

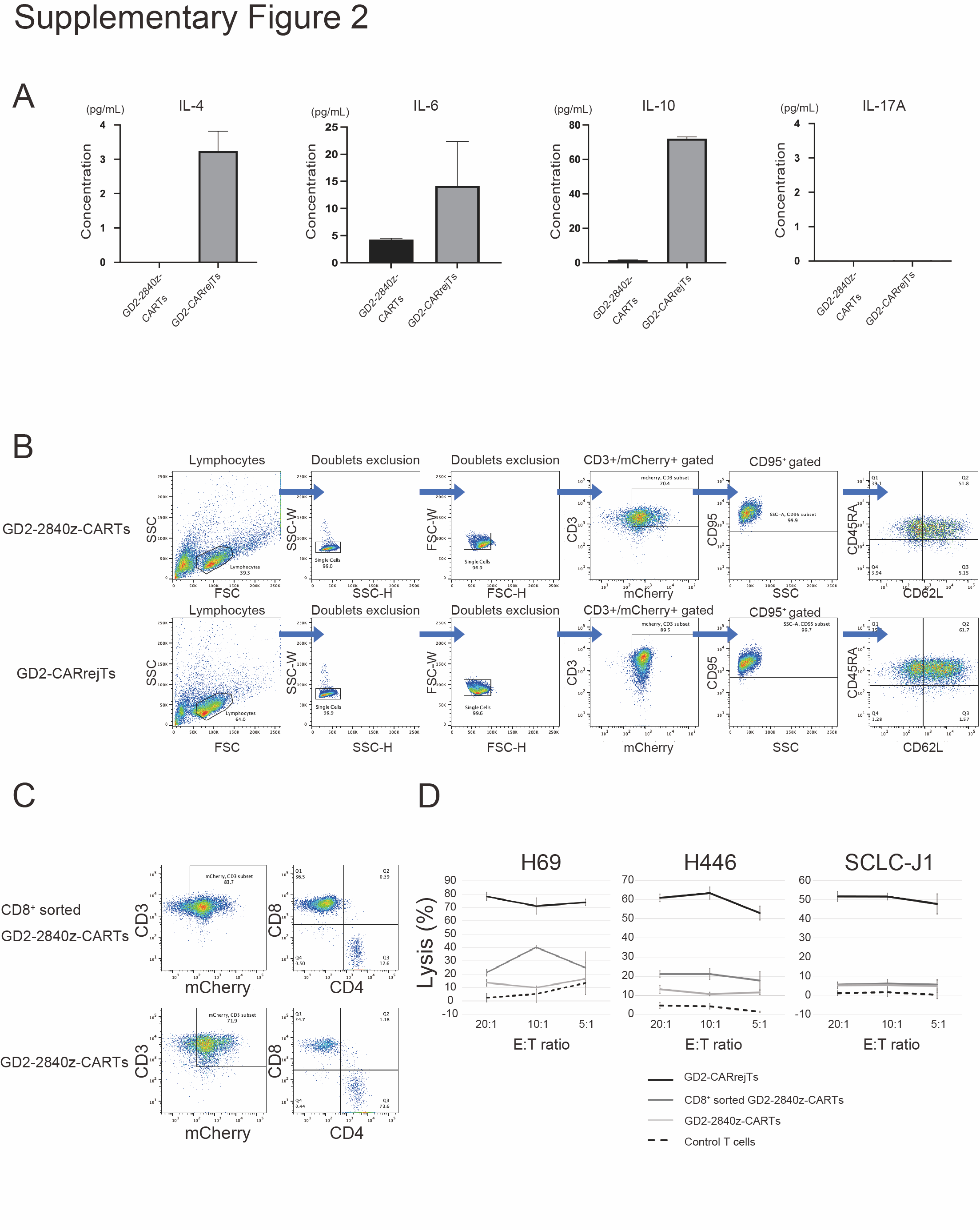
 Supplementary Figure 2. Functional and phenotype assays of GD2-CARTs and GD2-CARrejTs

(A) Cytometric bead assay (CBA) for measuring cytokines (IL-4, IL-6, IL-10, and IL-17A) produced by effector T cells (GD2-2840z-CARTs and GD2-CARrejTs) after 24 hours of coculture with SCLC-J1. Error bars represent ± SEM. (B) Gating strategy for flow cytometry of GD2-CARTs and GD2-CARrejTs. Lymphocytes were gated based on FSC and SSC, followed by doublet removal. To define the memory phenotype subsets of GD2-2840z-CARTs and GD2-CARrejTs, they were gated as CD3+/mCherry+ cells. (C) Flow cytometric analysis of GD2-2840z-CARTs and CD8^+^ sorted GD2-2840z-CARTs to evaluate CAR transgene expression and CD4/8 ratio. The plots represent 3 independent experiments. (D) *In vitro* ^51^Cr assays of GD2-CARrejTs, CD8^+^ sorted GD2-2840z-CARTs and GD2-2840z-CARTs against SCLC cell lines. Error bars represent ± SD. Data represent 2 independent triplicate experiments.
