# Supplementary material for: Rejuvenated iPSC-derived GD2-directed CART Cells Harbor Robust Cytotoxicity Against Small Cell Lung Cancer
Source: Cancer Res Commun. 2024 Mar 11;4(3):723–37. doi: 10.1158/2767-9764.CRC-23-0259 (PMC10926899; doi:10.1158/2767-9764.CRC-23-0259)
Supplement: Supplementary Figure 3 — Supplementary Figure S3 demonstrates the expression profiles of signature genes (including lineage, co-stimulation, co-inhibition, cytotoxic molecules, and transcription factors) in GD2-2840z-CARTs, GD2-CARrejTs, and control T cells through a heatmap. It also includes UMAP projections of CITE-seq data highlighting the distribution of CD3, CD4, and CD8 markers, and phenotypic markers specific to GD2-2840z-CARTs and GD2-CARrejTs. Additionally, a heatmap showcases the expression of chemokine receptor-related genes in these cell types, providing insights into their functional characteristics. [file crc-23-0259-s03.docx]

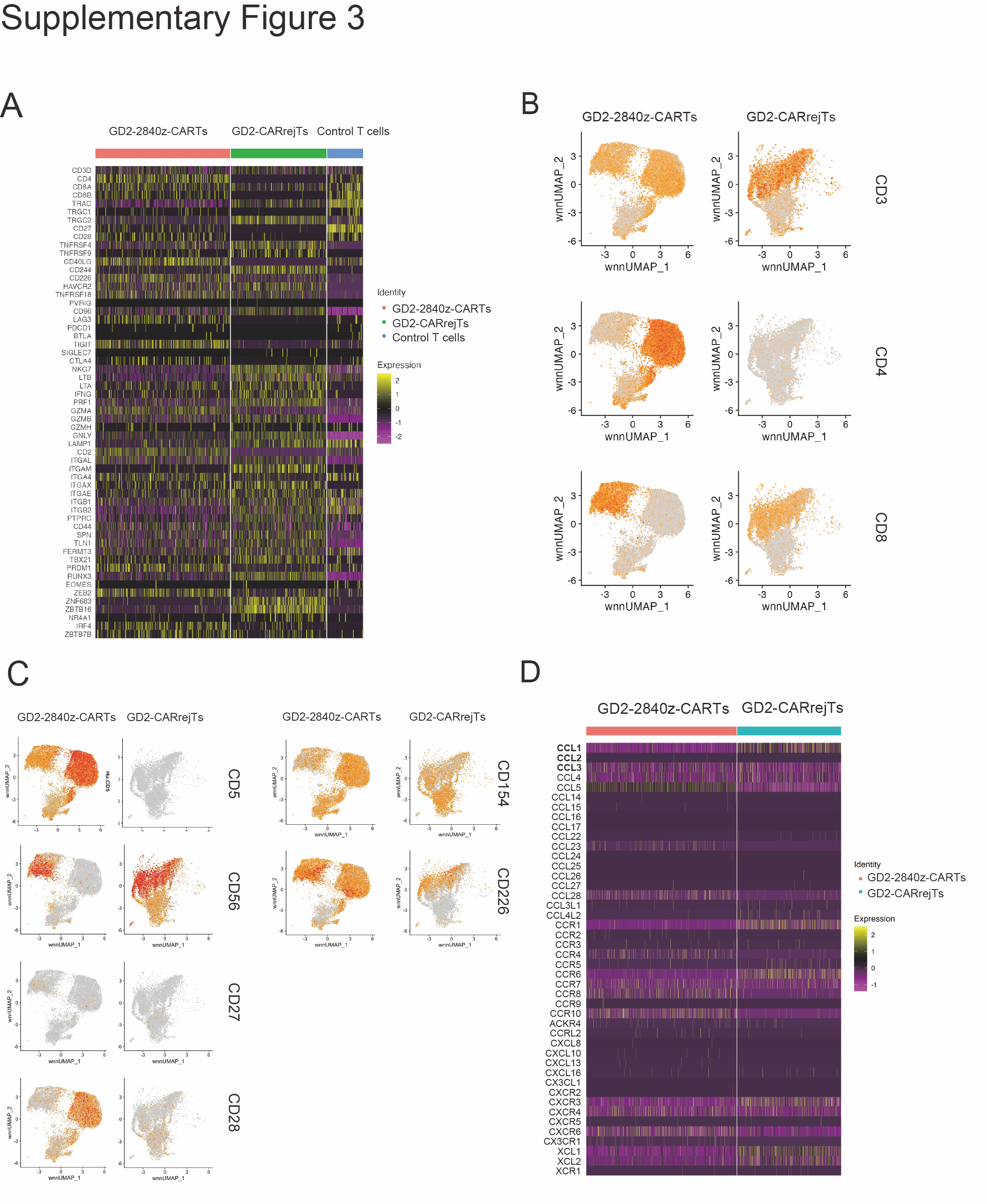


Supplementary Figure 3.

(A) Heatmap of signature genes (lineage, co-stimulation, co-inhibition, cytotoxic molecules, transcription factors) in GD2-2840z-CARTs, GD2-CARrejTs, and control T cells. (B) UMAP projection of CITE-seq data of CD3, CD4 and CD8. (C) UMAP projection of CITE-seq data (phenotypic markers) of GD2-2840z-CARTs and GD2-CARrejTs. (D) Heatmap of chemokine receptor related genes expressed in GD2-2840z-CARTs and GD2-CARrejTs.
