# Supplementary material for: Rejuvenated iPSC-derived GD2-directed CART Cells Harbor Robust Cytotoxicity Against Small Cell Lung Cancer
Source: Cancer Res Commun. 2024 Mar 11;4(3):723–37. doi: 10.1158/2767-9764.CRC-23-0259 (PMC10926899; doi:10.1158/2767-9764.CRC-23-0259)
Supplement: Supplementary Figure 4 — Supplementary Figure S4 presents flow cytometric analysis of PD-1, TIGIT, and CD226 expression on GD2-2840z-CARTs and GD2-CARrejTs after coculture with SCLC-J1 cells, based on three independent experiments. It also features real-time cell analysis (RTCA) showing tumor proliferation indices over 80 hours in various conditions: alone, in coculture with GD2-CARrejTs, and control T cells, with and without anti-TIGIT and anti-PD-1 antibodies. The data illustrate the effect of these treatments on tumor growth. [file crc-23-0259-s04.docx]

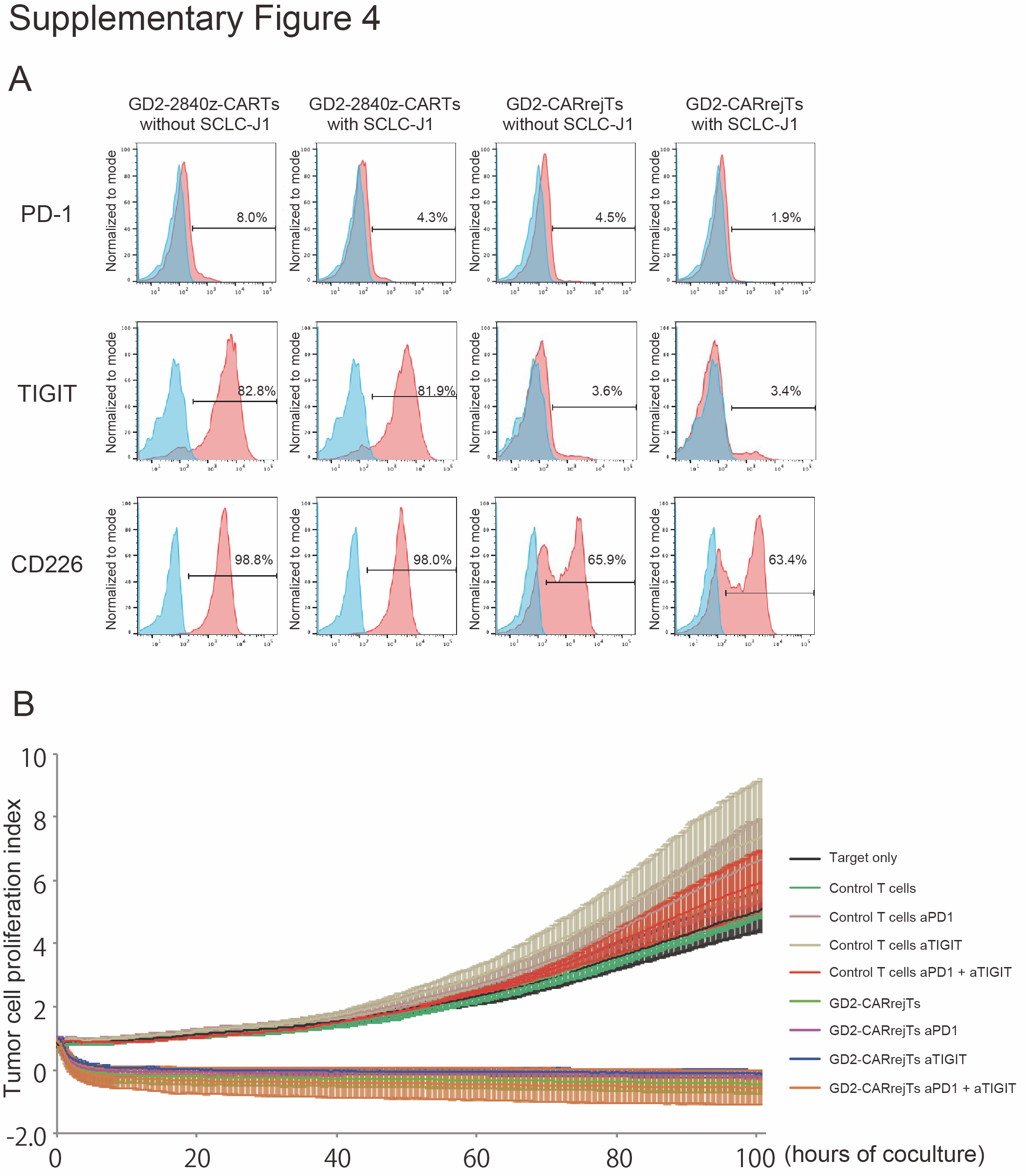


Supplementary Figure 4

(A) Flow cytometric analysis for PD-1, TIGIT, and CD226 expression on both GD2-2840z-CARTs and GD2-CARrejTs after 24 hours of coculturing with SCLC-J1 cells. The plots represent 3 independent experiments.

(B) RTCA continuous graphical output of tumor proliferation indices up to 80 hours alone and in coculture with GD2-CARrejTs and control T cells with / without anti-TIGIT (aTIGIT) and / or anti-PD-1 antibodies (aPD-1). Data were plotted and are shown as mean ± SD.
